# Strategic Modification of Gut Microbiota through Oral Bacteriotherapy Influences Hypoxia Inducible Factor-1α: Therapeutic Implication in Alzheimer’s Disease

**DOI:** 10.3390/ijms23010357

**Published:** 2021-12-29

**Authors:** Laura Bonfili, Chunmei Gong, Francesca Lombardi, Maria Grazia Cifone, Anna Maria Eleuteri

**Affiliations:** 1 School of Biosciences and Veterinary Medicine, University of Camerino, 62032 Camerino, Italy; chunmei.gong@unicam.it; 2 Department of Life, Health and Environmental Sciences, University of L’Aquila, 67010 L’Aquila, Italy; francesca.lombardi@univaq.it (F.L.); mariagrazia.cifone@univaq.it (M.G.C.)

**Keywords:** hypoxia-inducible factor-1α, Alzheimer’s disease, probiotics, microbiota, nitric oxide, prolyl hydroxylase 2

## Abstract

Dysbiosis contributes to Alzheimer’s disease (AD) pathogenesis, and oral bacteriotherapy represents a promising preventative and therapeutic opportunity to remodel gut microbiota and to delay AD onset and progression by reducing neuroinflammation and amyloid and tau proteins aggregation. Specifically, SLAB51 multi-strain probiotic formulation positively influences multiple neuro-chemical pathways, but exact links between probiotics oral consumption and cerebral beneficial effects remain a gap of knowledge. Considering that cerebral blood oxygenation is particularly reduced in AD and that the decreased neurovascular function contributes to AD damages, hypoxia conditioning represents an encouraging strategy to cure diseases of the central nervous system. In this work, 8-week-old 3xTg-AD and wild-type mice were chronically supplemented with SLAB51 to evaluate effects on hypoxia-inducible factor-1α (HIF-1α), a key molecule regulating host-microbial crosstalk and a potential target in neurodegenerative pathologies. We report evidence that chronic supplementation with SLAB51 enhanced cerebral expression of HIF-1α and decreased levels of prolyl hydroxylase 2 (PHD2), an oxygen dependent regulator of HIF-1α degradation; moreover, it successfully counteracted the increase of inducible nitric oxide synthase (iNOS) brain expression and nitric oxide plasma levels in AD mice. Altogether, the results demonstrate an additional mechanism through which SLAB51 exerts neuroprotective and anti-inflammatory effects in this model of AD.

## 1. Introduction

Gut dysbiosis is an established key player in the pathogenesis of Alzheimer’s diseases (AD) and, more generally, in age-related neurodegenerations. In fact, an increased concentration of proinflammatory bacteria in the gut is responsible for the enhanced secretion of lipopolysaccharides and amyloid peptides that can alter the intestinal permeability and the blood–brain barrier (BBB), consequently promoting neuroinflammation, oxidation, amyloid beta deposition, insulin resistance, and neuronal degeneration [1,2]. In AD, cerebral oxygen supply is impaired, contributing to neuroinflammation, plaque formation, neuronal death, and cognitive deficits with molecular mechanisms not well defined [3]. Neuroinflammation is associated to the overactivation of microglia, the resident innate immune cells that can trigger inflammatory signals and release proinflammatory cytokines, chemokines, and neurotoxic substances, such as matrix metalloproteinases, reactive oxygen species (ROS), and nitric oxide (NO).

No definitive cure is available for AD, but current treatments can temporarily reduce symptoms. Interestingly, oral bacteriotherapy is a new, appealing therapeutic approach to strategically modulate gut microbiota composition in order to counteract AD onset and progression, representing a debated issue [4]. In this context, the effects of the oral supplementation of SLAB51 multi-strain probiotic formulation were previously studied in a transgenic murine model of AD, demonstrating that this formulation can influence multiple neuro-chemical pathways, by increasing the plasma concentration of short chain fatty acids and neuroprotective gut hormones, restoring the impaired proteolytic pathways, triggering antioxidant mechanisms, and ameliorating energy homeostasis. Consequently, amyloid load and tau hyperphosphorylation decreased and behavioral tests demonstrated that memory integrity and attention improved in treated mice [5,6,7].

Studies have shown that hypoxia modulates amyloid precursor protein metabolism, reduces expression of enzymes that break down Aβ, and contributes to calcium-homeostasis dysregulation in neurons. Moreover, neuroinflammation is a key consequence of cerebral hypoxia and is strictly linked to AD pathogenesis [8]. 

HIF-1α is an oxygen regulated subunit that, in hypoxia, binds to HIF-1β subunit and forms a heterodimer with a role in glucose transport and metabolism, cell stress and survival, and the immune system [9]. Additionally, prolyl hydroxylase 2 (PHD2) is a negative regulator of HIF-1α and leads to proteasomal degradation of HIF-1α. Specifically, PHD2 regulates oxygen-dependent HIF-1α. degradation either in normoxia or following a short exposure to hypoxia [10]. It has been hypothesized that PHD inhibition may be part of a therapeutic strategy in AD because it can stabilize HIF with neuroprotective, anti-inflammatory and antioxidant consequences [11]. 

Considering that blood oxygenation is especially reduced in the hippocampus of AD subjects and that the decreased neurovascular function contributes to AD damages [12], hypoxia conditioning has been described as a promising strategy to cure diseases of the central nervous system [13,14]. 

Moreover, a recent study suggests that SLAB51 multi-strain probiotic formulation can reduce nitric oxide synthesis in intestinal cells, concluding that less oxygen consumption by intestinal cells implicates an increased oxygen supply to other organs, including the brain [15].

Hypoxia influences the pathogenesis of many neurodegenerative diseases, especially AD. The duration and extent of hypoxia determine its beneficial or detrimental effects; indeed, severe and/or chronic hypoxia can impair oxygen delivery and affect cellular metabolism, ATP production, and Ca^2+^ homeostasis, and it can lead to ROS formation and inflammation. Conversely, mild, moderate, or intermittent hypoxia has been shown to promote protective adaptations in the brain [11]. 

The present study aimed to evaluate the ability of SLAB51 oral treatment to induce HIF-1α stabilization, also analyzing the effect on PHD2 expression, along with inhibition of inducible nitric oxide synthase (iNOS) expression and activity in the brain of a well-known AD animal model (3xTg-AD mice), thus supporting the neuroprotective effects of probiotics oral supplementation on neuroinflammation in AD.

## 2. Results

### 2.1. SLAB51 Affected Nitric Oxide Related Pathways

**Figure 1:**
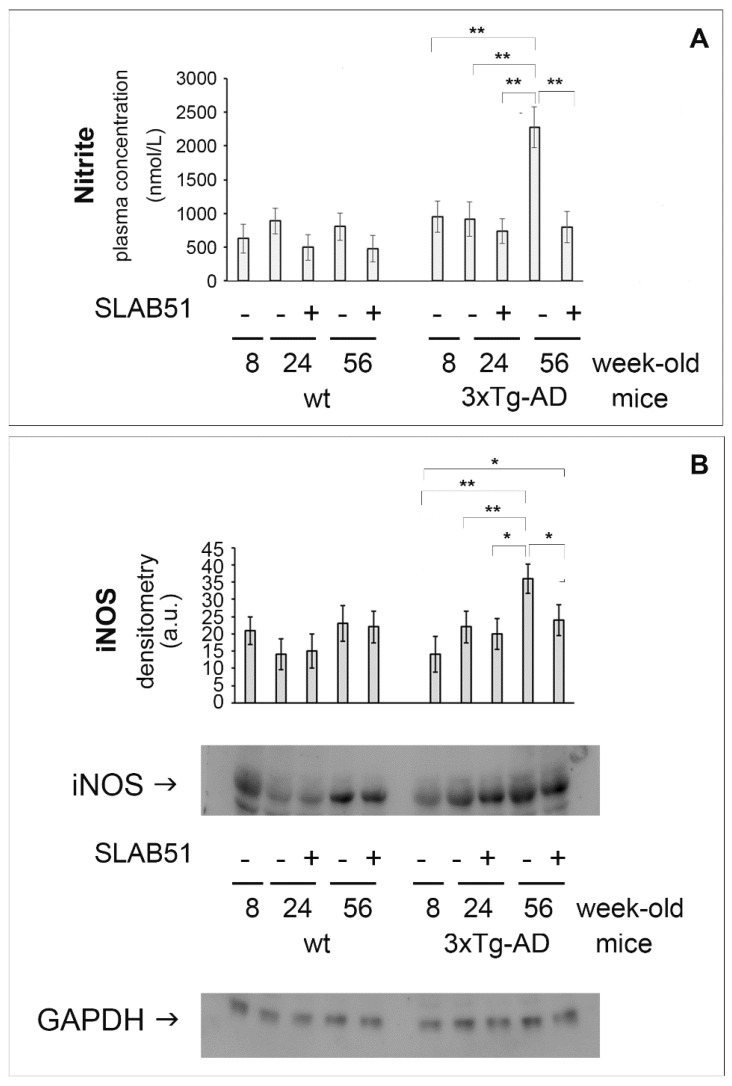
SLAB51 decreased plasma nitrite and iNOS cerebral expression in AD mice. (**A**): Nitrite plasma concentration (nmol/L) in 8-, 24-, and 56-week-old wt (left) and AD (right) mice administered with water or SLAB51. (**B**): Cerebral iNOS expression levels normalized by GAPDH measured in SLAB51-treated (+) and SLAB51-untreated (-) wt (left) and AD (right) mice. The densitometric analyses obtained from three separate blots and representative immunoblots are shown. Equal protein loading was verified by using an anti-GAPDH antibody. The detection was performed with an ECL Western blotting analysis system. Statistical significance compared to untreated 8-week-old mice and age-matched mice is indicated with asterisks (* *p* < 0.05, ** *p* < 0.01).

Nitric oxide (NO) production and oxidation are increased in the brains of AD subjects, contributing to neuronal cell loss and neurodegeneration [16]. As expected, nitrite plasma concentration and iNOS cerebral levels significantly increased in 56-week-old 3xTg-AD untreated animals with respect to younger transgenic mice. Interestingly, treatment with SLAB51 restored the plasmatic nitrite concentration and iNOS protein expression in the brain of these animals (Figure 1), indicating downstream positive effects of these changes on the compromised inflammatory and oxidative status in AD. Additionally, these results are in line with previously published evidence on the antioxidant effects of the probiotic formulation. Specifically, significantly decreased levels of 3-nitrotyrosine were detected in the brain of SLAB51-treated 3xTg-AD mice [6].

### 2.2. SLAB51 Restored HIF-1α Cerebral Expression in AD Mice

**Figure 2:**
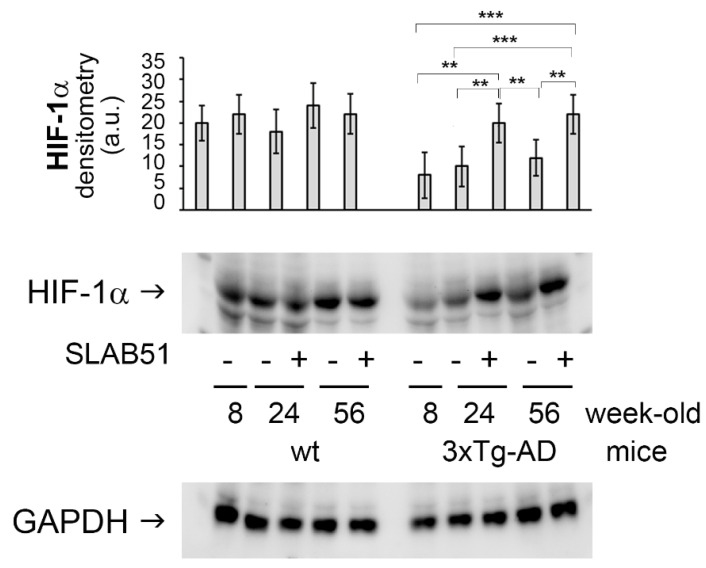
SLAB51 restored HIF-1α cerebral expression in AD mice. HIF-1α expression levels normalized by GAPDH measured in brain homogenates of SLAB51-treated and SLAB51-untreated wt (left) and AD (right) mice. The densitometric analyses obtained from three separate blots and representative immunoblots are shown. Equal protein loading was verified by using an anti-GAPDH antibody. The detection was performed with an ECL Western blotting analysis system. Statistical significance compared to untreated 8-week-old mice and age-matched mice is indicated with asterisks (** *p* < 0.01, *** *p* < 0.001).

Hypoxic responses are regulated by hypoxia-inducible transcription factor-1, with HIF-1α being the oxygen-regulated subunit. A decreased expression of HIF-1α in untreated 3xTg-AD mice compared to age-matched untreated wild type animals was observed. Chronic treatment with probiotics restored HIF-1α cerebral expression (Figure 2), suggesting a positive effect on oxygen homeostasis and glucose metabolism. In fact, HIF-1α regulates the activity of glucose transporters 1 and 3 (GLUT1 and GLUT3) that are crucial for brain glucose uptake [17]. These data are in agreement with a study demonstrating that HIF-1 levels are reduced in AD brains compared to age-matched controls, [18] resulting in decreased GLUT1 and GLUT3 levels and with abnormal tau hyperphosphorylation [3]. Interestingly, the increased brain expression of HIF-1α in SLAB51-treated 3xTg-AD mice is in agreement with previously published data demonstrating that these probiotics can ameliorate cerebral glucose uptake in AD mice by increasing the brain expression of GLUT1 and GLUT3 [7].

**Figure 3:**
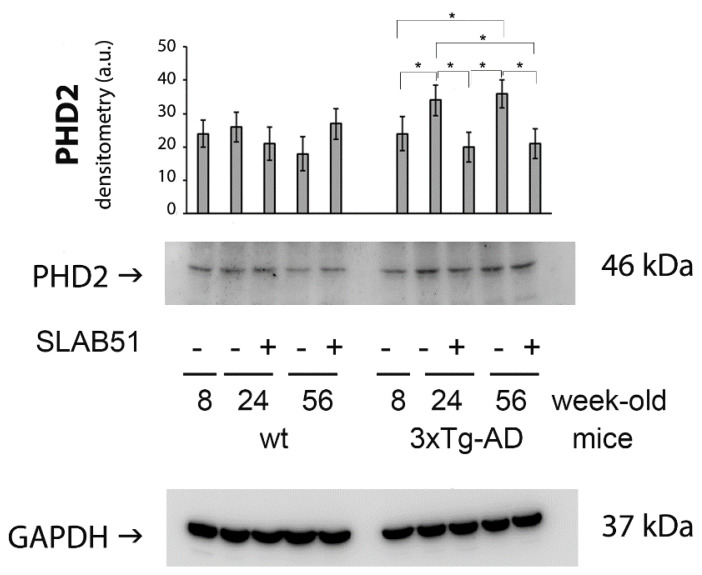
Decreased cerebral expression of PDH2 in treated AD mice. PHD2 expression levels normalized by GAPDH, measured in brain homogenates of SLAB51-treated and SLAB51-untreated wt (left) and AD (right) mice. The densitometric analyses obtained from three separate blots and representative immunoblots are shown. Equal protein loading was verified by using an anti-GAPDH antibody. The detection was performed with an ECL Western blotting analysis system. Statistical significance compared to untreated 8-week-old mice and age-matched mice is indicated with asterisks (* *p* < 0.05).

HIF-1α restored levels in treated mice correlated to the decreased brain expression levels of PHD2 upon SLAB51 chronic supplementation (Figure 3). In fact, PHD2 is known to regulate HIF-1α degradation, and the HIF/PHD2 pathway has been studied as a pharmaceutical target to promote the endogenous HIF transcriptional response for treatment of hypoxic human diseases [19].

## 3. Discussion

Gastrointestinal-tract microbiota-derived LPS is an important contributor to inflammatory neurodegeneration in the brain of AD patients [20], contributing to neuronal loss and impaired memory. Considering that, in AD subjects, specific cerebral regions are more susceptible to damages due to BBB dysfunction, impaired vascularization, and hypoxic insult [12], it is crucial to identify new strategies to improve neuronal function by modulating the expression level of key molecular agents that can finely modulate the gut–brain axis, reduce nitric oxide and ROS production, and counteract neuronal cell loss. In particular, during hypoxia the oxygen-regulated subunit HIF-1α forms a heterodimer with the constitutively expressed HIF-1β, influencing the transcription of important regulators of glucose homeostasis, cellular stress, inflammation, and apoptosis [21]. Moreover, HIF can induce genes involved in anaerobic metabolism, such as GLUT1 and GLUT3, and genes encoding glycolytic enzymes [22]. Additionally, HIF-1α is degraded in an oxygen dependent manner under regular oxygenated conditions through the hydroxylation of proline residues by prolyl hydroxylases, particularly PHD2. Several factors, such as ROS [23] and NO [24], can interfere with PHD activity finally influencing HIF-stability. In particular, HIF-1α and PHDs are directly regulated by S-nitrosylation, leading to stabilization of HIF-1α. Normoxic HIF-1 activity can be up-regulated through NO-mediated S-nitrosylation and stabilization of HIF-1α; however, during hypoxia, NO is inactivated by superoxide to form deleterious peroxynitrite, known to destabilize HIF-1α. [24].

SLAB51 multi-strain probiotic formulation is able to shift microbiota composition and counteract AD progression in transgenic mice [5], and a recent clinical study reported the effect of oral bacteriotherapy to increase nitric oxide synthesis in intestinal cells, with positive consequences in vital organs like the brain [15]. In the present work, the ability of the same probiotics to positively interfere with the HIF-1α/PHD2 pathway in 3xTg-AD mice was explored in order to clarify the incompletely understood mechanisms of action of SLAB51.

The brain expression of HIF-1α was reduced in AD mice with respect to age-matched wild type animals, indicating compromised inflammatory status [21] and immune response [25]. Interestingly, upon SLAB51 oral administration, restored HIF-1α brain levels suggest a positive effect on oxidative status and immune functionality, but also indicate a beneficial effect on glucose metabolism. In fact, HIF-1α regulates the activity of glucose transporters 1 and 3 (GLUT1 and GLUT3) that are crucial for brain glucose uptake [17]. These data are in agreement with a study demonstrating that HIF-1 levels are reduced in AD brains compared to age-matched controls [18], resulting in decreased GLUT1 and GLUT3 levels and with abnormal tau hyperphosphorylation [3]. The increase of HIF-1α in 24- and 56-week-old AD mice administered with SLAB51 corroborates previously published data showing that chronic treatment with the same probiotic formulation ameliorated glucose uptake in 3xTg-AD mice by increasing the brain expression of GLUT1 and GLUT3 [7]. Moreover, PHD2 is a negative regulator of HIF-1α which causes its hydroxylation, followed by polyubiquitination and proteasomal degradation [26]. Inhibition of PHD2 is a promising therapeutic approach because not only has pleiotropic neuroprotective effect as a consequence of HIF-1α. induction, but also exerts both antioxidant and anti-inflammatory actions [11]. Interestingly, the decreased expression of PHD2 reflects the upregulation of HIF-1α in treated animals. Furthermore, SLAB51 chronic administration counteracted the increase of nitrite plasma concentration and iNOS cerebral levels, indicating both an antioxidant and anti-inflammatory effect of SLAB51, in agreement with previously published data on the increased gut concentration of anti-inflammatory bacterial metabolites in 3xTg-AD mice treated with the same probiotic formulation [5] and with the ameliorated oxidative status in this animal model [6]. Specifically, we have previously observed decreased nitrotyrosine accumulations in the brains of 3xTg-AD mice treated with probiotics [6], and other authors demonstrated that iNOS is aberrantly increased in both neurons and glial cells of AD subjects [27] being structurally related to nitrotyrosine formation [28].

Potentially negative side effects of microbiome alterations [29] should be carefully evaluated [4] in future pre-clinical and clinical studies. In fact, microbiome structure and genetic and epigenetic factors can influence the final effect [30]. SLAB51 beneficially impacts gut microbiota in 3xTg-AD mice with multi-level mechanisms, and these data contribute to describe another molecular mechanism through which this specific probiotic formulation can counteract oxidation and inflammation and can improve energy metabolism in AD.

## 4. Materials and Methods

### 4.1. Animal Studies

AD triple-transgenic mice, B6;129-Psen1tm1Mpm Tg (APPSwe, tauP301L)1Lfa/J (named 3xTg-AD), and their respective wild-type animals were purchased from the Jackson Laboratory (Bar Harbor, ME, USA). 3xTg-AD mice contain three mutations associated with familial Alzheimer’s disease (APP, Tau MAPT P301L, and Presenilin-1 M146V). This reliable model of human AD displays both plaque and tangle pathology, with Aβ intracellular immunoreactivity detectable at three months of age and hyperphosphorylation of tau protein occurring by 12 to 15 months of age [31]. Experiments were in accordance with the guidelines laid down by the European Communities Council (86/609/ECC) for the care and use of laboratory animals and with a protocol approved by the Italian Ministry of Health (518/2018-PR). Mice were housed in Makrolon ® cages (4 animals per cage) in a temperature-controlled room (21 ± 5 °C) and 60% humidity on a 12 h light/dark inverted cycle (light was switched on at 8:00 p.m.) and maintained on laboratory diet (Mucedola Srl, Milano, Italy) with water ad libitum. Appropriate measures minimized pain and discomfort in experimental animals.

SLAB51 probiotic formulation was kindly provided by Prof. Claudio De Simone, MD (Ormendes SA, Jouxtens-Mézery, Switzerland, https://agimixx.net, accessed on 29 December 2021). SLAB51 contains eight different live bacterial strains: Streptococcus thermophilus DSM 32245, Bifidobacterium lactis DSM 32246, Bifidobacterium lactis DSM 32247, Lactobacillus acidophilus DSM 32241, Lactobacillus helveticus DSM 32242, Lactobacillus paracasei DSM 32243, Lactobacillus plantarum DSM 32244, and Lactobacillus brevis DSM 27961.

Eight-week-old AD male mice (*n* = 48) were divided in two groups: one administered with SLAB51 dissolved in water (*n* = 24), and the control group administered with water (*n* = 24). Simultaneously, 48 age-matched wild type (wt) mice were organized into wt control (*n* = 24) and wt-treated (*n* = 24) groups. The dosage of SLAB51 (200 bn bacteria/Kg/day) was determined by application of the body surface area principle [32]. The body weight was monitored during the treatment to ensure single-housed animals received the proper intake of treatment. Preliminary studies were performed to evaluate both viability and stability of the probiotic formulation upon solubilization in water at 21 ± 5 °C. The percentage of vital bacteria was determined by fluorescence microscopy, which revealed that 88% of the strains survived after 30 h under the above-mentioned conditions. Thus, probiotic drinking solution was freshly prepared every day. Eight mice per group were euthanized at 8, 24, and 56 weeks of age, and the tissues were properly collected and stored at −80 °C.

### 4.2. Preparation of Brain Homogenates

Upon sacrifice, mice brains were homogenized (1:5 weight/volume of buffer) in 50 mM Tris buffer, 150 mM KCl, 2 mM EDTA, pH 7.5. Homogenates were immediately centrifuged at 13,000× *g* for 20 min at 4 °C, and the supernatant was used. Protein concentration was measured through the Bradford protein assay [33].

### 4.3. Western Blot Analysis

Mice brain homogenates were analyzed through western blotting to measure the levels of inducible nitric oxide synthase (iNOS), hypoxia-inducible factor 1-α (HIF-1α), and prolyl hydroxylase 2 (PHD2). In detail, for each time point, brain homogenates (30-µg total protein) were resolved on a 10–12% sodium dodecyl sulphate-polyacrylamide gel electrophoresis (SDS-PAGE) and electroblotted onto polyvinylidene fluoride membranes for chemiluminescent western blotting. Molecular weight markers (6.5 to 205 kDa) were included in each gel. Glyceraldehyde-3-phosphate dehydrogenase (GAPDH) was used to check equal protein loading and to normalize the target proteins signal. The densitometric analysis has been conducted using Image J software as previously described [7].

### 4.4. Nitrite Level Assay

Nitrite quantitation in the plasma of treated and untreated AD and wt mice was performed with the Griess Reagent kit for nitrite determination (Molecular Probes, Leiden, The Netherlands) according to the manufacturer instruction.

### 4.5. Statistical Analysis

Statistical analysis of data was performed using Sigma-stat 3.1 software (SPSS, Chicago, IL, USA). For comparison between two means, Student’s unpaired test was used. For comparison of the mean values among the groups, upon Shapiro–Wilk normality test, a two-way ANOVA, followed by a Bonferroni post hoc test, was used. The results were expressed as mean values ± SD, as specified in figure legends. *p* values less than 0.05 were considered to be statistically significant and are indicated by one asterisk (*); two asterisks (**) indicates a *p* value of less than 0.01; three asterisks (***) indicates a *p* value of less than 0.001.

## Data Availability

The data presented in this study are available on request from the corresponding author.
